# FGFR2 mutations promote endometrial cancer progression through dual engagement of EGFR and Notch signalling pathways

**DOI:** 10.1002/ctm2.1223

**Published:** 2023-05-10

**Authors:** Garima Dixit, Jesus Gonzalez‐Bosquet, Joseph Skurski, Eric J. Devor, Erin B. Dickerson, Warren B. Nothnick, Priya D. Issuree, Kimberly K. Leslie, Thorsten Maretzky

**Affiliations:** ^1^ Inflammation Program University of Iowa Iowa City Iowa USA; ^2^ Department of Internal Medicine University of Iowa Iowa City Iowa USA; ^3^ Department of Obstetrics and Gynecology University of Iowa Iowa City Iowa USA; ^4^ Holden Comprehensive Cancer Center Roy J. and Lucille A. Carver College of Medicine, University of Iowa Iowa City Iowa USA; ^5^ Immunology Graduate Program University of Iowa Iowa City Iowa USA; ^6^ Department of Veterinary Clinical Sciences College of Veterinary Medicine University of Minnesota St. Paul Minnesota USA; ^7^ Masonic Cancer Center University of Minnesota Minneapolis Minnesota USA; ^8^ Animal Cancer Care and Research Program University of Minnesota St. Paul Minnesota USA; ^9^ Cell Biology and Physiology Center for Reproductive Sciences University of Kansas Medical Center Kansas City Kansas USA; ^10^ Division of Molecular Medicine Departments of Internal Medicine and Obstetrics and Gynecology The University of New Mexico Comprehensive Cancer Center University of New Mexico Health Sciences Center Albuquerque New Mexico USA

**Keywords:** a disintegrin and metalloprotease 10, a disintegrin and metalloprotease 17, endometrial cancer, epidermal growth factor receptor, fibroblast growth factor receptor 2, Notch

## Abstract

**Background:**

Mutations in the receptor tyrosine kinase gene *fibroblast growth factor receptor 2* (*FGFR2*) occur at a high frequency in endometrial cancer (EC) and have been linked to advanced and recurrent disease. However, little is known about how these mutations drive carcinogenesis.

**Methods:**

Differential transcriptomic analysis and two‐step quantitative real‐time PCR (qRT‐PCR) assays were applied to identify genes differentially expressed in two cohorts of EC patients carrying mutations in the *FGFR2* gene as well as in EC cells harbouring mutations in the *FGFR2*. Candidate genes and target signalling pathways were investigated by qRT‐PCR assays, immunohistochemistry and bioinformatics analysis. The functional roles of differently regulated genes were analysed using in vitro and in vivo experiments, including 3D‐orthotypic co‐culture systems, cell proliferation and migration protocols, as well as colony and focus formation assays together with murine xenograft tumour models. The molecular mechanisms were examined using CRISPR*/*Cas9‐based loss‐of‐function and pharmacological approaches as well as luciferase reporter techniques, cell‐based ectodomain shedding assays and bioinformatics analysis.

**Results:**

We show that common *FGFR2* mutations significantly enhance the sensitivity to FGF7‐mediated activation of a disintegrin and metalloprotease (ADAM)17 and subsequent transactivation of the epidermal growth factor receptor (EGFR). We further show that FGFR2 mutants trigger the activation of ADAM10‐mediated Notch signalling in an ADAM17‐dependent manner, highlighting for the first time an intimate cooperation between EGFR and Notch pathways in EC. Differential transcriptomic analysis in EC cells in a cohort of patients carrying mutations in the *FGFR2* gene identified a strong association between *FGFR2* mutations and increased expression of members of the Notch pathway and ErbB receptor family. Notably, FGFR2 mutants are not constitutively active but require FGF7 stimulation to reprogram Notch and EGFR pathway components, resulting in ADAM17‐dependent oncogenic growth.

**Conclusions:**

These findings highlight a pivotal role of ADAM17 in the pathogenesis of EC and provide a compelling rationale for targeting ADAM17 protease activity in FGFR2‐driven cancers.

## Graphical abstract

Graphical Abstract: Endometrial cell migration and proliferation depends on the metalloprotease ADAM17. FGFR2 mutations render endometrial cancer cells more sensitive to FGF7 stimulation. FGF7‐induced activation of FGFR2 prompts transcriptional reprogramming in FGFR2‐mutant expressing EC cells.

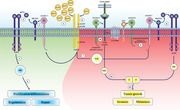

## 1 INTRODUCTION

Endometrial cancers (ECs) are neoplasms of the endometrium and represent one of the most diagnosed malignant tumours of the female genital tract.^1^, ^2^, ^3^ Molecular analyses have previously identified somatic mutations in the *fibroblast growth factor receptor 2* (*FGFR2*) in 30% of EC cell lines as well as in up to 16% of ECs^4^, ^5^, ^6^, ^7^ and are associated with advanced and recurrent diseases.^8^ Although various mutations of *FGFR2* in EC exist,^4^, ^6^, ^7^ the mechanistic basis for their potential tumorigenic activity in EC remains incompletely understood. FGF7/FGFR2‐induced cell migration of human keratinocytes depends on epidermal growth factor receptor (EGFR)‐mediated extracellular signal‐regulated kinase (ERK) signalling and a disintegrin and metalloprotease (ADAM) 17‐dependent proteolytic release (shedding) of the EGFR ligand heparin‐binding EGF‐like growth factor (HB‐EGF).^9^ In addition, ADAM17 plays a crucial role in various types of cancers.^10^, ^11^, ^12^, ^13^ These observations raise the possibility that mutations in the *FGFR2* could contribute to tumour growth and metastasis formation in EC by regulating ADAM17‐mediated EGFR/ERK signalling pathways that induce proliferation and transformation in epithelial cells of the endometrium. The main goal of the current study was to evaluate what role, if any, ADAM17 has in the activation of EGFR/ERK in ECs harbouring mutations in the *FGFR2* and in promoting their proliferation and growth in response to FGF7/FGFR2 signalling.

Utilizing isogenic cells as well as several EC cell lines that have previously been established from Type I cancers to model disease biology,^14^, ^15^ including *FGFR2*‐mutant MFE280,^16^ MFE296^16^ and AN3CA,^17^ as well as *FGFR2*‐wild type (WT) SKUT1B,^18^ Ishikawa^19^ and KLE,^20^ we show that the most common EC‐linked mutations in *FGFR2* (S252W and N550K^21^) significantly increase the sensitivity of FGF7 ligand–mediated ADAM17 activity, leading to increased shedding of HB‐EGF. Strikingly, FGF7‐dependent activation of mutant FGFR2 also led to the engagement of ADAM10‐mediated Notch signalling. We identify genetic programs uniquely altered in mutant *FGFR2*‐expressing (*FGFR2*‐mutant) ECs, suggesting that dysregulated ADAM17 activity as a consequence of *FGFR2* mutations elicits altered cellular signalling and transcriptional reprogramming to drive the malignant transformation of the endometrium. Our results may inform clinical decisions regarding personalized therapeutic modulation of EGFR/ERK and Notch signalling pathways in this high‐risk population and reveal the potential utility of ADAM17 inhibition in treating patients with EC and more broadly patients with other FGFR2‐dependent tumours.

## 2 MATERIAL AND METHODS

### 2.1 Cell lines and culture

AN3CA (#HTB‐111), SKUT1B (#HTB‐115), HEC1A (#HTB‐112), KLE (#CRL‐1622) and T HESCs (#CRL‐4003) cell lines were obtained from American Type Culture Collection (ATCC; USA) and cultured as recommended. The generation and culture procedure of immortalized human endometrial epithelial cells (EM‐E6/E7/TERT, EM) has been described previously.^22^ Briefly, EM‐TERT cells were maintained in Dulbecco's Modified Eagle Medium (DMEM)‐F12 (Gibco/Fisher Scientific, USA) supplemented with 10% heat‐inactivated foetal calf serum (FCS; Atlanta Biologicals, USA), 1% penicillin/streptomycin (PS; #15140122, Gibco/Fisher Scientific, USA) and insulin–transferrin–selenium–ethanolamine (ITS‐X; #51500056, Invitrogen, USA). 293FT (#R70007) cells were obtained from Thermo Fisher Scientific, USA and cultured in high‐glucose DMEM (Gibco/Fisher Scientific, USA), 500 µg/mL Geneticin (#10131035, Gibco/Fisher Scientific, USA), 10% FCS, 10% DMSO (#D2438, Millipore‐Sigma, USA), .1 mM MEM nonessential amino acids (#11140050, Gibco/Fisher Scientific, USA), 6 mM l‐glutamine (#25030081, Gibco/Fisher Scientific, USA), 1 mM MEM sodium pyruvate (#11360070, Gibco/Fisher Scientific, USA) and 1% PS. Ishikawa (#99040201), MFE296 (#98031101) and MFE280 (#98050131) cell lines were obtained from MilliporeSigma, USA and cultured as recommended. All cell lines were maintained in an incubator set at 37°C with a constant supply of 5% CO_2_. Cell lines were routinely tested for mycoplasma contamination. Opti‐MEM (# 31985070, Gibco/Fisher Scientific, USA) was used for cell starvation.

### 2.2 Patient tumour sample analysis

The institutional review board (IRB) of the UI approved the current study, including human subjects/materials on July 28, 2016 (IRB Number 201607815: ‘*Prediction Model for Risk Assessment in Endometrial Cancer*’) and on April 25, 2018 (IRB Number 201804817: ‘*Prediction Models in Ovarian Cancer’*).

### 2.3 RNA purification and sequencing in University of Iowa

The University of Iowa Department of Obstetrics and Gynecology maintains a Women's Health Tissue Repository (WHTR) containing more than 60 000 biological samples, including more than 2500 primary gynaecologic tumors.^23^ All tissues in the WHTR were collected under informed consent (IRB Numbers 200910784 and 200209010). Tumour samples were collected and reviewed by a board‐certified pathologist at the time of diagnosis and flash frozen. Specimens had less than 30% of necrosis. Of the 126 patients identified in the original EC panel, we were able to obtain 62 primary tumour EC samples with sufficient RNA yield and quality for analysis. Total cellular RNA was purified from primary tumour tissue using the mirVana (Thermo Fisher, USA) RNA purification kit following manufacturers’ instructions. Yield and quality of purified RNA were measured using a Trinean DropSense 16 spectrophotometer and an Agilent Model 2100 bioanalyzer. Only RNAs with an RNA integrity number^24^ greater than or equal to 7.0 were selected for RNA sequencing. RNA processing and sequencing have been described elsewhere.^25^, ^26^, ^27^ Briefly, equal mass total RNA (500 ng) from each qualifying tumour was fragmented, converted to cDNA and ligated to bar‐coded sequencing adaptors using Illumina TruSeq stranded total RNA library preparation (Illumina, San Diego, CA, USA). Molar concentrations of the indexed libraries were confirmed on the Agilent Model 2100 bioanalyzer, and libraries were then combined into equimolar pools for sequencing. The concentration of the pools was confirmed using the Illumina Library Quantification Kit (Kapa Biosystems, Wilmington, MA, USA). Sequencing was then carried out on the Illumina HiSeq 4000 genome sequencing platform using 150 bp paired‐end SBS chemistry. Library preparation and sequencing were performed in the Genome Facility of the University of Iowa Institute of Human Genetics (IIHG).

### 2.4 File pre‐processing and analysis

Sequence reads were mapped and aligned to the human reference genome (version hg38) using STAR, a paired‐end enabled algorithm.^28^ BAM files were produced after alignment. We used featureCounts to measure gene expression from BAM files.^29^ After the gene counts were generated, we used the DESeq2 package to import, normalize and prepare the data for analysis.^30^ BAM files for each sample were also used for mutation discovery and base‐calling against the human genome reference utilizing SAMtools and BCFtools.^31^ After filtering for duplicates, known non‐synonymous single‐nucleotide variants, and synonymous variants, results were annotated and classified with ANNOVAR and formatted to display the number of variants per gene and sample.^32^ To determine differences in gene expression between patients with and without *FGFR2* mutations, we used multiple two‐sample *t*‐test analyses. The significance level was at *p*‐value < .001 to account for multiple comparisons. Those differentially expressed genes were introduced in an enrichment pathway analysis using the *clusterProfiler* R package,^33^ which interrogates the Kyoto Encyclopedia of Genes and Genomes (KEGG) database.

### 2.5 The Cancer Genome Atlas (TCGA)

Patients with non‐endometrioid histology were excluded. Of those patients with Type I EC, or endometrioid EC, RNA‐seq data were downloaded. Same methods were used to measure gene expression from BAM files and to annotate and classify single‐nucleotide variants (see Section 2.4).

### 2.6 Expression vectors

The expression vectors for the alkaline phosphatase (AP)‐tagged HB‐EGF, betacellulin (BTC) inactive ADAM17E > A (EA A17) and WT ADAM17 (WT A17) have been described previously.^9^, ^34^ FGFR2 mutant constructs were generated by using full‐length FGFR2 cDNA as template.^35^ The QuikChange site‐directed mutagenesis kit (Stratagene, CA, USA) was used to generate the point mutations of FGFR2.

### 2.7 Growth factors and inhibitors

The metalloprotease inhibitor DPC 333 ((2*R*)‐2‐((3*R*)‐3‐amino‐3 (4‐[2‐methyl‐4‐quinolinyl) methoxy] phenyl)‐2‐oxopyrrolidinyl)‐*N*‐hydroxy‐4‐methylpentanamide)) (DPC) was a gift from Dr. Carl P. Blobel (Weill Cornell Medicine, Graduate School of Medical Sciences, NY, USA) and diluted in DMSO to the indicated concentrations. The following intracellular signalling inhibitors were used: AG1478 (#141438, Abcam, USA); DAPT (#D5942), SB202190 (#S7067), CRM197 (#D2189), Dasatinib (#CDS023389) and G1254023X (#SML0789) obtained from MilliporeSigma, USA; LY294002 (#154447‐36‐6) and U0126 (#109511‐58‐2) obtained from Calbiochem, USA and recombinant human FGF7 (# 251‐KG, R&D Systems, USA).

### 2.8 Transfection and ectodomain shedding assay

Cells were seeded in 12‐well cell culture plates to obtain 40%–60% confluency on the next day. Prior to transfection, cells were starved for at least 1 h in reduced serum medium (Opti‐MEM) and transiently transfected with either 1.5 µg/well FGFR2 WT or mutant‐plasmid (in case of EM cells) and/or 1 µg/well AP‐tagged HB‐EGF or BTC for at least 6 h as indicated using Lipofectamine (LF) 2000 (#11668019, Gibco/Fisher Scientific, USA) or for 48 h using FuGENE HD Transfection Reagent (#E2311, Promega). Post‐transfection, cells were replenished with the indicated growth medium and incubated overnight at 37°C, 5%, CO_2_. Cells were then serum starved for 4 h in Opti‐MEM, which was replaced with fresh Opti‐MEM with or without growth factors and/or inhibitors as indicated for 45 min to 1 h. Supernatants were collected and cells were lysed with lysis buffer (pH 9.5) containing Tris base (100 mM), NaCl (100 mM), MgCl_2_ (20 mM), 1–10 Phenanthroline (.5 M) and EDTA (.5 M) for 30 min at 4°C. Supernatants and cell lysates were loaded in triplicates on a 96‐well plate, and AP activity was measured by colourimetry at 405 nm.^36^ The ratio of AP activity in the supernatant to total AP activity in the cell lysate plus supernatant was calculated from three identically prepared wells and averaged. The ratio reflects the activity of a metalloprotease towards a given AP‐tagged receptor or ligand.

### 2.9 Small interfering RNA transfection

For transient silencing of ADAM17 (#HSS110434, #HSS110435, #HSS186181) and HB‐EGF (#HSS102973, #HSS102974, #HSS102975), cells were grown to 40%–60% confluency and transfected with 20 pMol stealth small interfering (si) RNA duplex (Thermo Fisher Scientific, USA) using LF RNAi MAX transfection reagent (#13778150, Thermo Fisher Scientific, USA) according to manufacturer's instructions. Random stealth siRNA duplexes (High GC duplex) served as controls (#12935100, Thermo Fisher Scientific, USA). After 3‐day incubation at 37°C, the cells were serum starved in Opti‐MEM for 8 h and used in scratch wound‐healing assays. siADAM17‐transfected cultures were assayed for constitutive or induced HB‐EGF shedding 48 h after siRNA delivery. Afterwards, the cells were processed for quantitative real‐time PCR (qRT‐PCR) to analyse knockdown efficiency.

### 2.10 Total RNA isolation and quantitative real‐time PCR

Cells were subjected to total RNA isolation via RNeasy Plus Mini Kit (#74106, Qiagen, USA). RNA quantity and quality were measured on NanoDrop ND‐1000 spectrophotometer (Thermo Fisher Scientific, USA). cDNA was synthesized using 2 µg of total RNA following manufacturer's protocol (ProtoScript First Strand cDNA Synthesis Kit, #E6300L, NEB, USA). Synthesized cDNA was used to perform qRT‐PCR for all sets of selected genes using a commercial mastermix (PerfeCTa SYBR Green FastMix, #95071012, Quanta bio, USA). Human *actin* and *gapdh* primer sets were used as internal controls to normalize and relative expression levels of target genes were calculated by using the ΔΔ*Ct* method.^37^ Pre‐verified primers (KiCqStart SYBR Green Primers) were purchased from MilliporeSigma, USA.

### 2.11 Lentiviral production and transduction

To either knock‐down ADAM17, HB‐EGF, FGFR2, or FGF7, guide RNA specific to human‐ADAM17 (Top Sg CACCGATCTAATATCCAGCAGCATT, Bottom Sg AACAATGCTGCTGGATATTAGATC), human‐HB‐EGF (Tog Sg CACCGATTCGGCCGAAGGAGCTACG; Bottom Sg AAACCGTAGCTCCTTCGGCCGAATC) human FGFR2 (Top Sg: CACCGCTTAGTCCAACTGATCACGG; Bottom Sg: AAACCCGTGATCAGTTGGACTAAGC) and human‐FGF7 (Top Sg CACCGGTCGAACACAGTGGTACCTG; Bottom Sg: AAACCAGGTACCACTGTGTTCGACC) were cloned into lentiviral expression vector (LentiCRISPRv2‐mCherry) (#99154, Addgene, USA), sequence verified and co‐transfected into 293FT cells with the lentiviral packaging plasmids VsVG and Δ8.91 (#8454, Addgene, USA). In brief, 3 × 10^6^ 293FT cells were seeded into a 10‐cm culture dish the day before transfection. For each 10‐cm culture dish, the following DNA was diluted in 250 µL of Opti‐MEM: 7.5 µg of lentiviral vector, 2.25 µg of VsVG and 5.25 µg of Δ8.91. Separately, 11.25 µL of LF 3000 (# L3000008, Invitrogen, USA) was diluted into 250 µL of Opti‐MEM, vortexed and incubated at room temperature (RT) for 5 min. After incubation, the DNA and LF 3000 mixtures were combined to a final volume of 500 µL, briefly vortexed and incubated at RT for 30 min. During this incubation, the culture medium was replaced with 12 mL of DMEM containing 10% FCS. The transfection mixture was then added dropwise to the 10‐cm culture dish and incubated overnight. The next day, the medium was replaced with fresh DMEM‐F12 containing 10% FCS. Supernatants from the packaging reaction were collected at 48 and 72 h, respectively. Pooled supernatants were concentrated in 100 kDa Amicon Ultra‐15 centrifugal filter units (#UFC910024, MilliporeSigma, USA). Initially, viral transduction was optimized to achieve 90% transfection in EC cells, and 40 µL of optimized viral titre was used to transfect EC cells seeded in a 6‐well plate. Cells transduced with viral titre containing empty vector and packaging plasmids served as controls. Cells were grown for the next 48 h and then replenished with the fresh culture medium. mCherry‐positive cells were quantified and sorted by FACS (Becton Dickinson LSR II). Normally, more than 50%–60% of cells were mCherry positive. Sanger sequencing and loss‐of‐function assays were performed to validate CRISPR*/*Cas9‐mediated gene deletion.

### 2.12 Western blot analysis

For total protein isolation, cells were washed with PBS and lysed at 4°C in lysis buffer (pH 7.4) containing 1% triton, 150 mM NaCl, 1 mM EDTA, 1 mM 1–10 phenanthroline, protease inhibitor, 10 mM NaF, 5 mM β‐glycero‐P and 2 mM NaVO_3_. Insoluble material was removed by centrifugation at 13 000 rpm for 5 min. Protein was quantitated using Bradford reagent (#B6916, MilliporeSigma, USA), resolved by SDS–PAGE and transferred to nitrocellulose membranes (#66485, BioTrace NT Nitrocellulose Transfer Membrane, USA) and probed with antibodies against, phospho‐p44/42 MAPK (#9101; ERK1/2, Thr202/Tyr204) and p44/42 MAPK (#9102; ERK1/2) from Cell Signaling Technology, USA. Protein bands were detected using ECL chemifluorescent reagent (#RPN2106, GE HealthCare, USA) and imaged on Image Studio v4.0 in the LI‐COR Odyssey FC infrared imager.

### 2.13 Dual‐luciferase reporter assay

pHES1(467)‐luc (#41723, Addgene, USA) and pRL‐TK (#E2241, Promega, USA) plasmids have been described previously.^38^ *Renilla* luciferase dual assay was performed using Pierce *Renilla* Luciferase Dual Assay Kit (#16185, Thermo Fisher Scientific, USA) following the manufacturer's instructions. Briefly, cells were seeded in 96‐well plates to be 90% confluent at the time of transfection. Cells were washed with PBS and starved for 4 h before transfection. Experimental plasmid (pHES1(467)‐luc) and reporter plasmid (pRL‐TK) were diluted separately in Opti‐MEM and incubated for 5–10 min at RT. Transfection mixture was prepared by mixing diluted plasmids with LF 2000 and incubated for 30 min at RT. After 48 h of transfection, the treatment medium was replenished with a fresh defined medium and incubated for 48 h. Post‐transfection, cells were incubated with stimulators and/or inhibitors as indicated for 24 h at 37°C, 5%, CO_2_. Freshly prepared mixture of luciferin (substrate) and buffer was added in each treated well and measured for red *firefly* luciferase activity at 640 nm LP filter and green *Renilla* luciferase activity at 525 nm BP filter (SpectraMax i3x Multi‐Mode Detection Platform). The luciferase reporter plasmid (pRL‐TK) activity was used to control for transfection efficiency.

### 2.14 TUNEL assay

Apoptotic cells of paraffin‐embedded tumour sections were detected using the HRP‐DAB TUNEL staining kit (#206386, Abcam, Cambridge, UK) according to the manufacturer's instructions and counterstained with .5% methyl green. Stained slides were imaged under a bright‐field microscope (EVOS XL Core, Invitrogen).

### 2.15 In vitro scratch wound‐healing assay

For in vitro scratch wound‐healing assays cells were seeded in 12‐well plates and cultured until they reached 100% confluence. A scratch wound was introduced with a 200 µL pipette tip. After washing with PBS, the cells were incubated with or without the indicated inhibitors or stimuli. After 24 h, cells at the same positions along the scratch wound were photographed using a bright‐field microscope (EVOS XL Core, Invitrogen), and NIH ImageJ software was used for quantification.

### 2.16 Cell proliferation assay

Cells were seeded in the defined medium at 1 × 10^4^ cells per well in 96‐well cell culture plates. Next day, cells were starved for 4 h in Opti‐MEM before treating with stimulators and/or inhibitors as indicated. Cellular proliferation was assessed after 72 h in the presence or absence of 50 ng/mL recombinant FGF7. Cells were washed with PBS before adding a final conc. of .5 mg/mL of thiazolyl blue tetrazolium bromide solution to each well (#M2128, Sigma‐Aldrich, USA). Cells were incubated for next 4 h at 37°C. Medium was removed, and plates were dried upside down at RT overnight. Dried crystals were dissolved in DMSO. After 30 min of incubation at 37°C, absorbance was measured at 570 nm on an ELISA plate reader (SpectraMax Plus 384). All absorbance values were corrected to a medium‐only well (served as blank) and normalized to the values at day 0.

### 2.17 Focus formation assay

Cells were plated at a density of 2.5 × 10^5^ cells in 12‐well culture dishes and allowed to adhere overnight before treatment with Opti‐MEM or full medium containing either 10% FCS or recombinant FGF7 (50 ng/mL). Cells were grown for 2 weeks and harvested for qRT‐PCR analysis.

### 2.18 Soft agar colony formation assay

Cells at a density of 500 cells per well were suspended in a top layer of defined medium (DMEM‐F12 or MEM) containing 5% calf serum and .3% Select agar (#A5054, MilliporeSigma, USA) and plated over bottom layer consisting of defined medium with 10% calf serum and .6% select agar in 24‐well cell culture plates. FGF7 and inhibitors were added as indicated in the figure legends. Fresh treatment solutions were added at least twice a week. After 2 weeks of incubation, cell colonies were counted from 10 fields photographed under a bright‐field microscope (EVOS XL Core, Invitrogen). Colony forming efficiency and surviving fraction were calculated as described earlier.^39^

### 2.19 Transwell cell invasion analysis

The cell invasion assay was carried out using a 24‐well transwell insert with a 3.0‐µm pore size polycarbonate membrane (#3415, CoStar), following the manufacturer's protocol. Transwell membranes were precoated with 100 µL of a 1:8 DMEM‐diluted Matrigel matrix basement membrane (#354234, Corning) and incubated at 37°C for 6 h. Cells were suspended in 100 µL of full‐medium and seeded on top of the Matrigel layer at a density of 3 × 10^4^ cells per well and incubated with or without DPC (2.5 µM) and recombinant FGF7 (50 ng/mL) at 37°C for 48 h. After 48 h incubation at 37°C, cells remaining at the upper surface of the membrane were removed with cotton swabs. The cells on the lower surface of the transwell membrane represent the invasive cells. After fixation with 4% paraformaldehyde and staining with crystal violet solution, cells that passed through the filter were photographed in five random visual fields under a light microscope (EVOS XL Core, Invitrogen). The relative invasion was calculated as the ratio of invading cells over the cell number normalized on day 2 of the growth curve.

### 2.20 3D‐Organotypic co‐culture

Organotypic cultures were performed following a modified protocol as described previously.^40^ Briefly, transwell polycarbonate membrane cell culture inserts (#3414, CoStar, USA) were placed in a deep 6‐well plate (#355467, Corning, USA) precoated with 2.2 mg/mL high concentration rat tail collagen (#354249, Corning, USA) and incubated at 37° for 30 min. T HESCs at the conc. of 3 × 10^5^ cells per mL in Matrigel matrix basement membrane (#354234, Corning, USA) were seeded and allowed to grow for a week. MFE296 cells (1 × 10^7^ cells per mL assay medium) were suspended in a medium containing 10% FCS. A volume of 50 µL of the cells were added to each well, forming a triangle pattern of droplets and incubated for 1 h at 37°C before adding the epidermalization medium. After 1 week of epidermalization, tissues were fixed in fresh 4% paraformaldehyde overnight at 4°C. Fixed cells were placed first in 10% and then in 20% sucrose in PBS for 2 and 4 h, respectively, and then in 30% sucrose in PBS, overnight. Cells were embedded in optimal cutting temperature compound (#4583, Tissue‐Tek, Sakura) and flash frozen in liquid nitrogen. Cryosections were prepared using a Thermo HM 525 cryostat (Thermo Fisher Scientific, USA) and transferred on precleaned Superfrost Plus Microscope Slides (#12‐550‐15, Thermo Fisher Scientific, USA). Haematoxylin and eosin (H&E) staining was performed using an automated slide stainer. Slides were air‐dried for 2–4 h and imaged under a light microscope (EVOS XL Core, Invitrogen).

### 2.21 Engraftment and molecular characterization of xenograft tissues

NOD.Cg‐Prkdcscid Il2rgtm1Wjl/SzJ (NSG; strain #005557) immunodeficient mice were purchased from the Jackson Laboratory, USA and maintained in a specific pathogen‐free environment and fed ad libitum. All procedures involving mice were approved by the Institutional Animal Care and Use Committee (IACUC) of the University of Iowa, IA, USA. For the generation of cell‐derived xenografts, 5 × 10^4^ viable tumour cells resuspended in 100 µL of complete media without FCS were mixed with an equal volume of Matrigel and inoculated into the flanks of 6–8‐week‐old female mice and monitored for growth. DPC was administered intraperitoneally at a dose of 6 mg/kg.^41^ Tumour volumes were measured using a regular scale. The greatest longitudinal diameter (length) and the greatest transverse diameter (width) were measured.^42^ Tumours were surgically resected at a size of 200–400 mm^3^. Tumour volumes were estimated by the modified ellipsoidal formula: volume = 1/2 (length × width^2^).^43^ DPC was administered as indicated in the figure legends. Tumours were fixed overnight in 10% neutral buffered formalin.

### 2.22 Tumour tissue processing, histology and immunohistochemistry

Tumour tissues were harvested immediately after sacrificing the NSG mice, fixed with 10% neutral buffered formalin for 18–24 h, processed and embedded in paraffin. The samples were sectioned at 6 µm using microtome (RM2135, Leica, USA). H&E staining was performed following standard protocols. For IHC, antigen retrieval was carried out by boiling the sections in citrate buffer (pH 6) for 10 min, followed by cooling at RT for 1 h. To eliminate endogenous peroxidases, tissues were treated in methanol containing 3% H_2_O_2_ for 30 min. Tissues were permeabilized with .1% Triton X‐100 for 30 min. Sections were further blocked in 10% goat serum for 30 min followed by incubation with primary antibodies at 4°C overnight. Tissues were washed with PBS and subsequently incubated with secondary antibody at 37°C for 1 h. Antibodies against Ki67 (#16667, Abcam), HES1 (D6P2U; #11988), Phospho‐p44/42 MAPK (ERK 1/2; #4370S) and mTOR (7C10; #2983) were purchased from Cell Signaling Technology, USA. Stained slides were imaged under a bright‐field microscope (EVOS XL Core, Invitrogen). For IHC scoring, both intensity and percentage of positive cells were considered. A total of 10 microscopy fields were reviewed in each section. Tumour cells with brown cytoplasm and/or nucleus or membrane were considered positive. Percentage of stained tumour cells is represented as IHC staining score.

### 2.23 Statistical analysis

Data were analysed using GraphPad Prism v8.0 or v9.0 software. Values are expressed as mean ± standard error of the mean of at least three independent experiments unless otherwise indicated. Statistical significance was determined by multiple‐comparison tests for in vitro experiments as described in the figure legends. Statistical comparisons between more than two groups were conducted by two‐way ANOVA. A *p*‐value <.05 was considered statistically significant.

## 3 RESULTS

### 3.1 FGF7‐stimulated endometrial cell migration and proliferation depends on ADAM17 and EGFR

**FIGURE 1:**
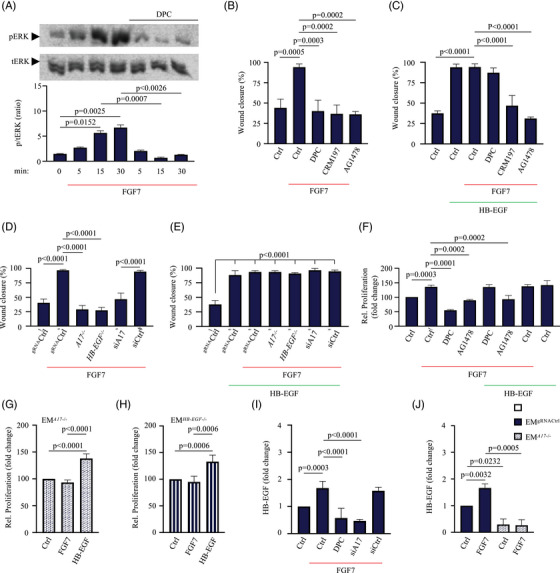
FGF7‐stimulated endometrial cell migration and proliferation depends on a disintegrin and metalloprotease 17 (ADAM17)‐mediated crosstalk between fibroblast growth factor receptor 2 (FGFR2) and epidermal growth factor receptor/extracellular signal‐regulated kinase (EGFR/ERK)‐signalling: (A) representative western blot analysis of ERK phosphorylation at different time points is shown for human endometrial EM‐E6/E7/TERT (EM) cells. *n* = 3 for densitometric quantification of ERK phosphorylation. Data are expressed as mean ± standard error of mean (SEM); one‐way ANOVA with Tukey's multiple comparison test. *p*‐Values indicate significant differences in FGF7‐stimulated ERK phosphorylation compared to unstimulated (vehicle) or DPC333 (DPC)‐treated cells at a given time point; (B–J) EM, *ADAM17*‐deficient (A17^−/−^) EM (EM^A17−/−^; D, E, G and J) or *heparin‐binding EGF‐like growth factor* (*HB‐EGF*)‐deficient (HB‐EGF^−/−^) EM (EM^HB‐EGF−/−^; D, E and H) were treated with or without FGF7 (50 ng/mL) or HB‐EGF (50 ng/mL) in the presence or absence of DPC (2.5 µM), CRM197 (10 µg/mL), AG1478 (10 µg/mL), small interfering RNA (siRNA) against ADAM17 (siA17; 20 pMol) or control siRNA (siCtrl; 20 pmol), as indicated (D, E and I); (B–E) a cell‐free area was introduced with a 200 µL pipette tip, and micrographs were taken at 0 and 24 h after scratch wounding. Quantification of the results of three separate scratch‐wound assays, with assays performed in duplicates, are shown. Data are expressed as mean ± SEM; one‐way ANOVA with Tukey's multiple comparison test. *p*‐Values indicate significant differences in FGF7‐ (B and D) and FGF7/HB‐EGF‐stimulated (C and E) scratch wound healing compared to unstimulated (B–E), inhibitor‐ (B and C) or siRNA‐treated cells (D and E); (F–H) quantification of the results of three separate proliferation assays, with assays performed in triplicates, of EM (F), EM*^A17^* ^−/−^ (G) or EM*^HB‐EGF^* ^−/−^ cells (H). Data are expressed as mean ± SEM; one‐way ANOVA with Tukey's multiple comparison test. *p*‐Values indicate significant differences in FGF7‐ (F) or HB‐EGF‐stimulated (G and H) cell proliferation compared to unstimulated (F–H) or inhibitor‐ (F) or FGF7‐treated cells (G and H); (I and J) effect of ADAM17 inactivation on HB‐EGF shedding. EM^siA17^ and EM^siCtrl^ (I), EM^gRNACtrl^ or EM*^A17^* ^−/−^ (J) cells were transfected with the alkaline phosphatase (AP)‐tagged ADAM17‐substrate HB‐EGF in the presence or absence of 2.5 µM DPC and stimulated with 50 ng/mL FGF7 for 45 min. Three independent experiments performed in triplicates. Data are expressed as mean ± SEM; one‐way ANOVA with Tukey's multiple comparison test (I) or two‐way ANOVA with Tukey's multiple comparison test (J). *p*‐Values indicate significant differences in shedding in FGF7‐treated cells compared with vehicle‐treated controls (Ctrl; I and J) or compared with inhibitor‐treated cells (I) or compared with EM*^A17^* ^−/−^ (J).

To determine whether FGFR2‐dependent phosphorylation of ERK (pERK) in endometrial cells requires activation of a metalloprotease, we tested how the hydroxamate metalloprotease inhibitor DPC 333 (DPC^41^) affects ERK phosphorylation at different time points after addition of FGF7 (50 ng/mL) to EM‐E6/E7/TERT (EM) cells, a human endometrial cell line with normal epithelial characteristics.^22^ Following the addition of FGF7, ERK phosphorylation was observed within 5 min and persisted for at least 30 min (Figure 1A). In contrast, the FGF7‐induced pERK was prevented by DPC in EM cells, even as early as 5 min after the addition of FGF7 (Figure 1A). This suggests that FGFR2 stimulated ERK phosphorylation by the activation of a metalloprotease, and not through an intracellular signalling pathway. To assess the functional relevance of metalloprotease‐dependent EGFR/ERK signalling in FGF7‐stimulated endometrial cells, we performed in vitro scratch wound healing assays with EM cells in the presence or absence of DPC, CRM197, which selectively inactivates the human form of HB‐EGF,^44^ or AG1478, a potent and specific inhibitor of EGFR signalling.^45^ Vehicle‐treated (Ctrl) EM cells did not repair scratch wounds after 24 h, whereas treatment with FGF7 led to the complete closure of the wound (Figure 1B, Figure S1A). FGF7‐stimulated the migration of EM cells could be blocked by DPC, CRM197 or AG1478 (Figure 1B, Figure S1A). The inhibition of FGF7‐dependent cell migration by DPC could be rescued by addition of human HB‐EGF (Figure 1C, Figure S1B), which is known to require processing by the membrane‐anchored metalloprotease ADAM17.^34^, ^46^, ^47^, ^48^, ^49^ However, HB‐EGF did not overcome the inhibition by CRM197 or AG1478, as these block binding of HB‐EGF to the EGFR or its activation, respectively (Figure 1C, Figure S1B). The requirement of the EGFR‐ligand HB‐EGF and a metalloprotease for FGF7‐stimulated migration of endometrial cells raised the possibility that ADAM17 is a critical intermediate in the signalling pathway between FGF7/FGFR2 and EGFR/ERK. To test this and to further confirm the requirement for HB‐EGF, we generated EM cell lines deficient for either *ADAM17* (EM*^A17^* ^−/−^) or *HB‐EGF* (EM*^HB‐EGF^* ^−/−^) using CRISPR*/*Cas9 technology. We also treated EM cells with anti‐ADAM17 short interfering RNA (siRNA, Figure S1C). Genetic deletion of either *ADAM17* (*A17* ^−/−^) or *HB‐EGF* (*HB‐EGF* ^−/−^) as well as anti‐ADAM17 siRNA (siA17) treatment blocked FGF7‐stimulated cell migration of EM cells, whereas treatment with control siRNA (siCtrl) or transduced with control guide RNA vector (^gRNA^Ctrl) did not (Figure 1D, Figure S1D). HB‐EGF could rescue the defect in cell migration caused by *ADAM17* or *HB‐EGF* deletion in FGF7‐stimulated cells (Figure 1E, Figure S1E). Moreover, the proliferation of EM cells over a time course of 72 h was increased in the presence of FGF7 (Figure 1F), whereas the FGF7‐stimulated proliferation of DPC or AG1478‐treated EM cells was significantly reduced (Figure 1F). The proliferation of DPC‐treated EM cells could be stimulated by HB‐EGF, demonstrating that EGFR‐dependent proliferation was not affected (Figure 1F). Similarly, FGF7‐stimulated proliferation was reduced in *ADAM17* (Figure 1G) or *HB‐EGF*‐deficient EM cells (Figure 1H) but could be rescued by the addition of HB‐EGF (Figure 1G,H).

To provide additional insights into the mechanism underlying the FGF7‐stimulated release of EGFR ligands, we evaluated the shedding of AP‐tagged HB‐EGF from EM cells.^9^, ^36^ We observed a significant increase in the shedding of HB‐EGF from EM cells stimulated with 50 ng/mL FGF7 that was abolished in DPC‐treated as well as anti‐ADAM17 siRNA‐treated EM cells (Figure 1I). To further confirm that FGFR2 stimulates the shedding of HB‐EGF by activating ADAM17, we performed similar experiments in *ADAM17‐deficient* EM cells. When *ADAM17‐deficient* EM cells were transfected with HB‐EGF, FGF7 was unable to stimulate HB‐EGF shedding (Figure 1J). These results demonstrate that the FGF7/FGFR2‐dependent activation of EGFR/ERK signalling in EM cells requires the stimulation of ADAM17 and the release of HB‐EGF.

### 3.2 FGFR2 mutations render EC cells more sensitive to FGF7 stimulation

**FIGURE 2:**
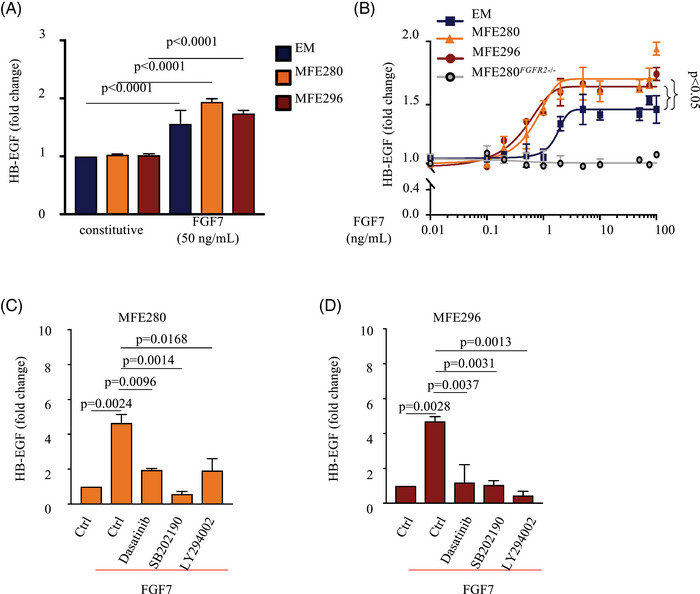
Endometrial cancer (EC)‐linked fibroblast growth factor receptor 2 (FGFR2) mutants increase the sensitivity of FGF7 ligand–induced a disintegrin and metalloprotease 17 (ADAM17) activity: (A) the alkaline phosphatase (AP)‐tagged ADAM17‐substrate heparin‐binding EGF‐like growth factor (HB‐EGF) was transfected into E6/E7/TERT (EM), MFE280 and MFE296 cells. Constitutive and FGF7‐stimulated (50 ng/mL) shedding of HB‐EGF into the supernatant was measured after 45 min. Three independent experiments performed in triplicates. Data are expressed as mean ± standard error of mean (SEM); two‐way ANOVA with Tukey's multiple comparison test. *p*‐Values indicate significantly increased shedding in FGF7‐treated cells compared with vehicle‐treated controls (Ctrl); (B) activation of ADAM17‐mediated shedding of HB‐EGF by FGF7 was determined in MFE280 and MFE296 cells compared with EM as well as MFE280*^FGFR2^* ^−/−^ cells. Three independent experiments performed in triplicates. Data are expressed as mean ± SEM; two‐way ANOVA showed a significant main effect of *FGFR2* gene status; (C and D) effect of signalling inhibitors on HB‐EGF shedding from FGF7‐stimulated MFE280 and MFE296 cells. Constitutive and FGF7‐stimulated shedding was assessed either without further additions or in the presence of 10 µM of the Src‐family kinase inhibitor Dasatinib, the p38 MAPK inhibitor SB202190 (10 µM) or the PI3‐kinase inhibitor LY294002 (10 µM). Three independent experiments performed in triplicates. Data are expressed as mean ± SEM; one‐way ANOVA with Tukey's multiple comparison test. *p*‐Values indicate a significantly increase in shedding of FGF7‐treated cells compared with inhibitor or vehicle‐treated cells.

Previous studies have shown that mutations in the *FGFR2* can elicit changes in FGFR2 ligand binding affinity and tyrosine kinase activity.^21^, ^50^ To test whether mutations in *FGFR2* alter the sensitivity of EC cells to FGF7/FGFR2‐mediated activation of ADAM17, we utilized some of the most widely used cell lines for EC research.^51^ We performed cell‐based shedding assays in the presence of FGF7 and measured the release of AP‐tagged HB‐EGF as a read‐out for ADAM17 activation. First, we treated WT FGFR2‐expressing (*FGFR2*‐WT) EM as well as mutant *FGFR2*‐expressing (*FGFR2*‐mutant) MFE280 and MFE296 EC cells with a dose of 50 ng/mL FGF7. Both EM cells and *FGFR2*‐mutant EC cells showed a similar increase in the release of HB‐EGF into the cell supernatant in response to FGF7 (Figure 2A). When we treated these cells with different FGF7 concentrations as low as .01 ng/mL, we observed that *FGFR2*‐mutant EC cell lines displayed a lower activation threshold for the stimulation of ADAM17, relative to *FGFR2*‐WT, suggesting that such a gain‐of‐function effect could contribute to EC progression (Figure 2B). The shedding of HB‐EGF in MFE280 and MFE296 cells (EC_50_ = .696 ± .29 ng/mL and EC_50_ = .457 ± .11 ng/mL) was about three‐to‐four times more sensitive to FGF7 stimulation compared with *FGFR2*‐WT EM cells (EC_50_ = 1.849 ± 1.71 ng/mL). Conversely, CRISPR*/*Cas9‐edited *FGFR2*‐deficient MFE280 cells (MFE280*^FGFR2^* ^−/−^) showed no ADAM17‐mediated release of HB‐EGF in response to FGF7 stimulation (Figure 2B).

To elucidate the downstream signalling pathways involved in the FGF7/FGFR2‐induced activation of ADAM17 in *FGFR2*‐mutant EC cells, we examined how different inhibitors of intracellular signalling affected the FGF7‐stimulated shedding of HB‐EGF (Figure 2C,D). We found that the Src‐family kinase inhibitor Dasatinib as well as the p38 MAP‐kinase inhibitor SB202190 and PI3‐kinase inhibitor LY294002 significantly reduced FGF7‐stimulated shedding of HB‐EGF. These results suggest that the activation of ADAM17 by FGFR2 in *FGFR2*‐mutant EC cells depends on Src, p38 MAP‐kinase and PI3‐kinase activity.

### 3.3 FGFR2‐mutant and WT endometrial tumours utilize distinct downstream signalling networks

**FIGURE 3:**
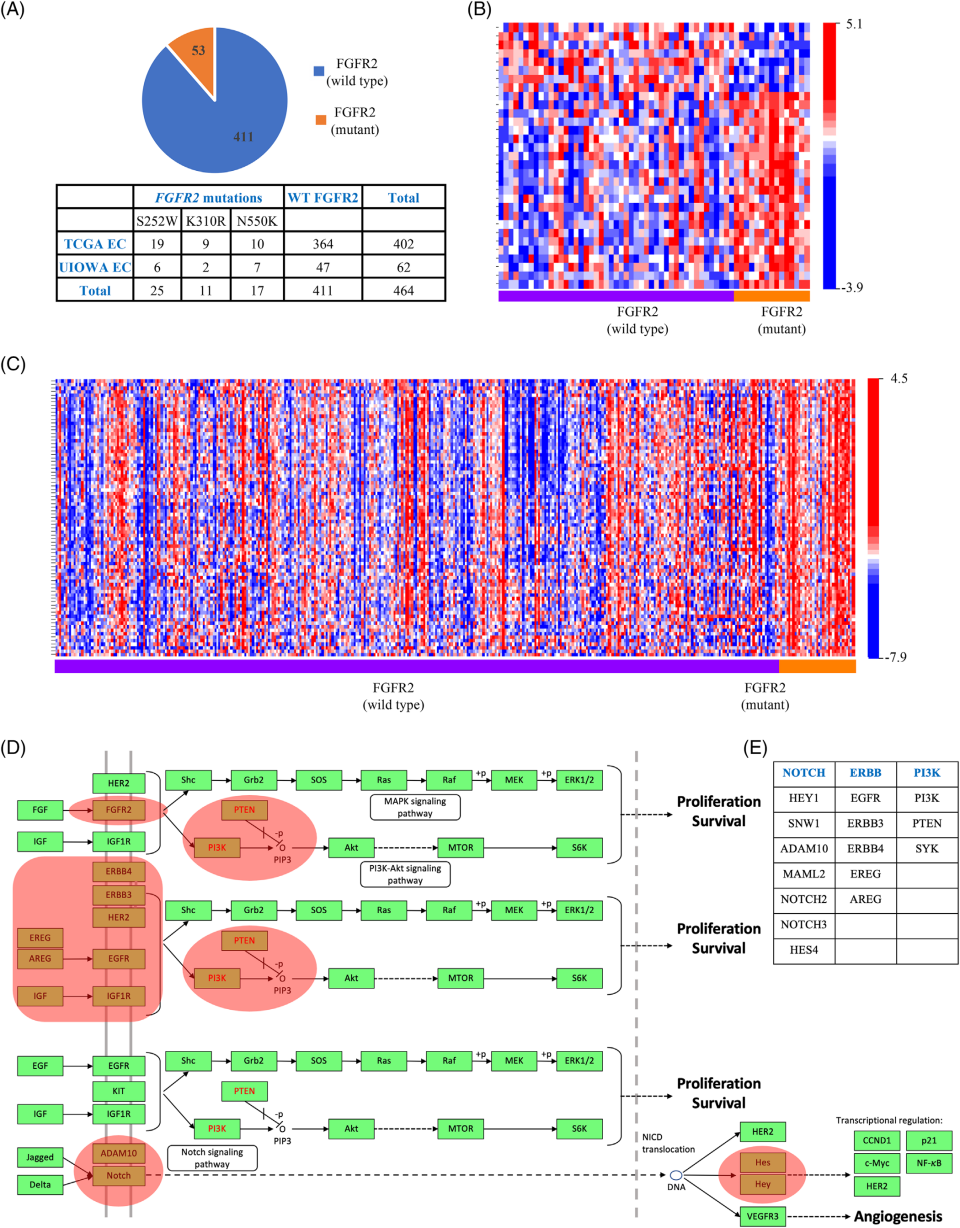
Transcriptional changes in *FGFR2*‐mutant endometrial cancer (EC): (A) proportion of *fibroblast growth factor receptor 2* (*FGFR2*)‐mutant tumours in a combined cohort of EC from The Cancer Genome Atlas (TCGA) and the University of Iowa (UIOWA): A total of 53 out of 464 patients with EC had *FGFR2*‐mutant tumours; (B and C) heat maps depicting differentially expressed genes between UIOWA (B) and TCGA (C) *FGFR2*‐wild type and *FGFR2*‐mutant ECs; (D) top canonical pathways, including ‘ErbB signalling’ and ‘Notch signalling’, represented in the results of the Kyoto Encyclopedia of Genes and Genomes (KEGG) pathway analysis; (E) the most prominent genes in these pathways are associated with the epidermal growth factor receptor (EGFR), PI3K and Notch pathways.

Previous studies have shown that increased EGFR signalling can lead to altered gene expression programs that contribute to oncogenic transformation and EC formation.^52^, ^53^, ^54^ To unbiasedly assess whether *FGFR2*‐mutant ECs display increased EGFR activity resulting from aberrant FGFR2‐associated ADAM17, we comparatively profiled the transcriptomes of 464 endometrial tumours (402 The Cancer Genome Atlas [TCGA] and 62 the University of Iowa), including 53 *FGFR2*‐mutant tumours (25 S252W, 11 K310R and 17 N550K‐mutants) and 411 *FGFR2*‐WT tumours (Figure 3A, Figure S2A,B). Hierarchical cluster analysis revealed unique gene expression signatures characteristic of each genetic tumour type (Figure 3B,C). To identify genetic programs specific to mutant FGFR2, we subjected the list of genes differentially expressed in *FGFR2*‐mutant tumours to KEGG pathway analysis and found the significant enrichment of genes prominently linked to ‘cancer’, ‘reproductive system disease’, ‘cell cycle regulation’ and ‘inflammatory responses’. Intriguingly, the top canonical pathways included ‘ErbB signalling’ and ‘Notch signalling’ (Figure 3D). The most prominent ‘regulators’ linked to these pathways included *EGFR*, *PI3K*, *NOTCH2* and *NOTCH3* (Figure 3E). These results suggest that transcriptional nodes related to EGFR and Notch signalling are significantly dysregulated in *FGFR2*‐mutant EC patients.

### 3.4 Transformed focus formation in FGFR2‐expressing EC cell lines depends on FGF7/FGFR2 activation

**FIGURE 4:**
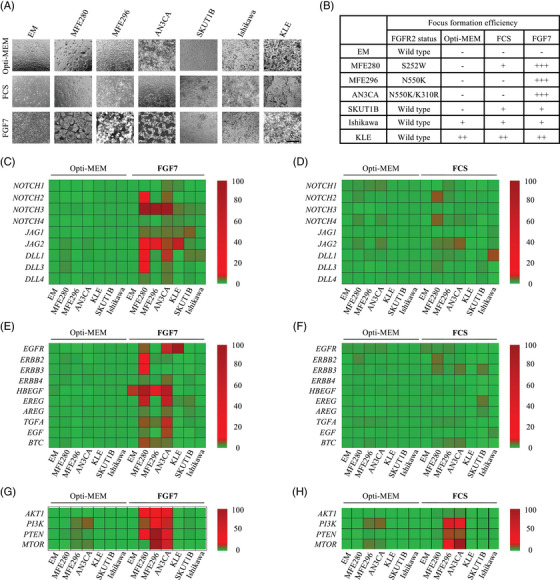
FGF7‐induced stimulation of mutant‐fibroblast growth factor receptor 2 (FGFR2) leads to Notch, epidermal growth factor receptor (EGFR) and AKT‐PI3K pathway gene activation in endometrial cancer (EC) cells: (A) representative images displaying focus forming efficiency of human endometrial EM‐E6/E7/TERT (EM), MFE280, MFE296, AN3CA, SKUT1B, Ishikawa and KLE cells grown in full medium containing 10% foetal calf serum (FCS) or serum‐reduced medium (Opti‐MEM) with or without 50 ng/mL FGF7 for 2 weeks (scale bar: 100 µm); (B) table displaying relative focus forming efficiency of cells in part (A). Quantification of results of three separate colony forming assays, each performed in duplicates is shown. Images of at least 10 random microscope fields were evaluated; (C–H) total RNA from cells in part (A) was harvested and processed by quantitative real‐time PCR (qRT‐PCR) to monitor mRNA expression of the indicated Notch, EGFR and PI3K pathway genes in the presence of FGF7 (C, E and G) or FCS (D, F and H). *n* = 3; each treatment performed in duplicates. mRNA levels of genes were normalized to *β‐actin* and *gapdh* mRNA and expressed relative to their corresponding mRNA levels in untreated EM cells. The mean of side‐by‐side replicates using double‐gradient colourmap with largest value set to 100, baseline value set to 3 and smallest value set to 0 is shown. Upregulated genes are displayed in red. The brightness of each colour corresponded to the magnitude of the difference when compared with average value.

Cells that contain a transforming oncogene can grow without contact inhibition that limits cell density and, on a confluent monolayer of cells, will form dense, raised foci.^55^ To determine how mutant FGFR2 expression could affect the loss of density‐dependent growth arrest in ECs, we performed focus formation assays with EC cell lines expressing WT FGFR2 (SKUT1B, Ishikawa, and KLE) or mutant FGFR2 (MFE280, MFE296 and AN3CA). We first compared how reduced serum medium (Opti‐MEM) or the addition of FCS or FGF7 affected their ability to produce foci after reaching confluence (Figure 4A,B; a higher magnification of Figure 4A is shown in Figure S2C). With the exception of MFE280 cells, which showed very limited capacity (+) to produce foci in the presence of FCS, Opti‐MEM‐cultured or FCS‐treated *FGFR2*‐mutant EC cells did not produce foci (−) even after 2 weeks in culture (Figure 4A, top and middle row). In contrast, FGF7‐treated *FGFR2*‐mutant EC cells manifested a malignant phenotype (++/+++) with a loss of density‐dependent growth inhibition, resulting in increased cellular packing and piling up of cells (Figure 4A, bottom row). When we conducted similar experiments with the three *FGFR2*‐WT EC cell lines, we found that focus formation in these cells did not depend on FGF7 stimulation. WT FGFR2‐expressing SKUT1B and Ishikawa cells formed foci independent of media conditions, whereas KLE cells only formed foci in the presence of either FGF7 or FCS. As expected, no focus formation was observed in EM cells (Figure 4A). Thus, in summary, these results suggest that focus formation in FGFR2‐mutant EC cells depends on FGF7‐mediated FGFR2 activation.

### 3.5 FGF7‐induced activation of FGFR2 prompts transcriptional reprogramming in FGFR2‐mutant expressing EC cells in vitro

To interrogate whether RNA‐sequencing transcriptomic signatures in EC patient samples mirror transcriptomic changes in EC cells, we next evaluated the expression of Notch (Figure 4C,D), ErbB (Figure 4E,F) and PI3K (Figure 4G,H) signalling components by two‐step qRT‐PCR in EC cell lines following FGF7‐induced focus formation (Figure 4A). We quantified a total of 23 transcripts related to these 3 pathways. All of the cell lines used in our study elicited only low levels of transcripts in the presence of reduced serum medium (Opti‐MEM; Figure 4C–H; Table S1). The comparison between Opti‐MEM alone and FGF7‐treated cells revealed that a total of 15 mRNAs were significantly increased in at least 1 of the 3 tested *FGFR2*‐mutant EC cell lines by more than 5‐fold (*p* < .05, Figure 3C,E,G). In MFE280 cells, at least a 5‐fold expression increase was found in 14 genes, including *NOTCH2* (5.31 ± .19) and *NOTCH3* (8.34 ± .12), *jagged canonical notch ligand 2* (*JAG2*) (8.00 ± .03), *delta like canonical notch ligand* (*DLL) 1* (8.57 ± .05) and *DLL3* (7.09 ± .32), *ERBB2* (5.97 ± .10), *ERBB3* (7.46 ± .16), *HBEGF* (11.53 ± .12), *epiregulin* (*ERE*G; 8.56 ± .03), *BTC* (8.26 ± .24), transforming growth factor alpha (*TGF*A; 8.66 ± .44) as well as *AKT1* (11.90 ± .81), *phosphoinositide 3‐kinase* (*PI3*K; 14.68 ± 1.00) and *phosphatase and tensin homolog* (*PTEN*; 12.09 ± .72) (Table S1). For MFE296 cells, a total of nine genes, including *NOTCH3* (6.81 ± .56), *JAG2* (9.18 ± .61), *HBEGF* (6.12 ± .63), *TGFA* (5.75 ± .56), *BTC* (14.16 ± 1.19) as well as *AKT1* (11.88 ± .18), *PI3K* (13.03 ± .410), *PTEN* (10.16 ± .33) and *mechanistic target of rapamycin kinase* (*MTOR*; 9.08 ± .20) were found to be at least twofold upregulated (Table S1). Our analysis also revealed at least a twofold upregulation of 12 genes in AN3CA cells (Table S1). In addition, EM cells showed a notable increase of only *HBEGF* expression (9.01 ± .55) in response to FGF7 treatment (Figure 4E), which is consistent with the HB‐EGF‐dependent and FGF7‐induced cell migration and proliferation of EM cells (Figure 1B–F). Although the moderate elevation of some PI3K pathway components was observed in the three *FGFR2*‐mutant EC cell lines tested in the presence of FCS supplementation (Figure 4H), none of the *FGFR2*‐WT cell lines tested, including EM cells appeared to significantly induce Notch, ErbB or PI3K pathways regardless of the media conditions (Figure 4C–H). These results further support the existence of distinctly triggered downstream responses in *FGFR2*‐mutant ECs.

### 3.6 Notch is a transcriptional target of aberrant FGFR2 function in EC

**FIGURE 5:**
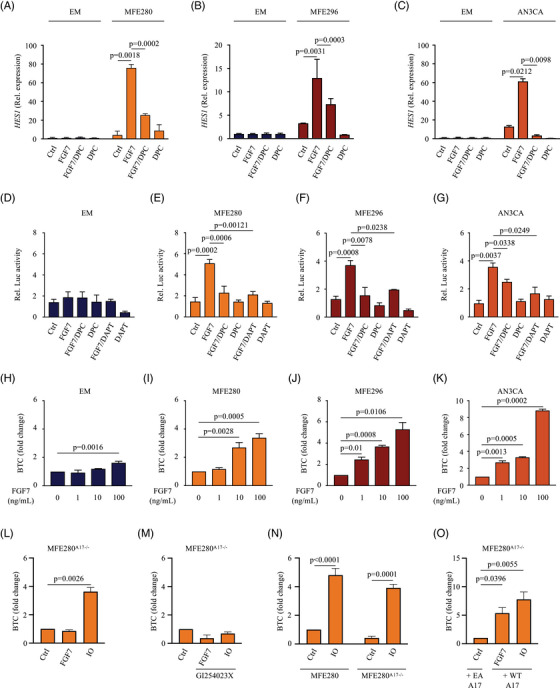
FGF7/Fibroblast growth factor receptor 2 (FGFR2) signalling drives metalloprotease‐dependent activation of Notch in *FGFR2*‐mutant endometrial cancer (EC) cells: (A–C) to measure FGF7/FGFR2‐mediated metalloprotease‐dependent Notch activation, *HES1* mRNA expression was determined in E6/E7/TERT (EM), MFE280, MFE296 or AN3CA cells treated with FGF7 (50 ng/mL) with or without DPC333 (DPC; 2.5 µM). Three independent experiments performed in triplicates. Data are expressed as mean ± standard error of mean (SEM); two‐way ANOVA with Tukey's multiple comparison test. *p*‐Values indicate significant differences in FGF7‐stimulated *HES1* gene expression compared to unstimulated or FGF7/DPC‐treated cells; (D–G) luciferase assay of *HES1* promoter activity in FGF7‐treated EM, MFE280, MFE296 or AN3CA cells with or without DPC or DAPT, respectively. pHES1(467)‐luc was used to drive firefly luciferase expression. Relative luciferase activity (Rel. Luc activity) represents pHES1(467)‐luc firefly luciferase activity divided by basal level of pRL‐TK Renilla firefly luciferase activity, with wild type *Renilla* luciferase reporter activity as the standardized internal control for transfection efficiency. Three independent experiments performed in triplicates. Data are expressed as mean ± SEM; one‐way ANOVA with Tukey's multiple comparison test. *p*‐Values indicate significant differences in FGF7‐stimulated *HES1* promoter activity compared to unstimulated or inhibitor‐treated cells; (H–O) to measure the activation of ADAM10 by FGF7, EM (H), MFE280 (I), MFE296 (J), AN3CA (K) or MFE280^A17−/−^ (L–O) cells were transfected with the alkaline phosphatase (AP)‐tagged ADAM10‐substrate betacellulin (BTC; H–O) or BTC and the inactive ADAM17E > A (EA A17), or wild type ADAM17, respectively (O), and treated with or without 1–100 ng/mL FGF7 (H–K), 50 ng/mL FGF7 (L–M) or 2.5 µM ionomycin (IO; L–O) in the presence or absence of .2 µM GI254023X. Three independent experiments performed in triplicates. Data are expressed as mean ± SEM; one‐way ANOVA with Dunnett's test (H–M) or two‐way ANOVA with Tukey's multiple comparison test (N and O). *p*‐Values indicate significantly increased shedding in FGF7‐ (H–K and O) or IO‐ (L, N and O) treated cells compared with vehicle‐treated controls (Ctrl).

To validate our observation that FGFR2 activation induces Notch signalling specifically in *FGFR2*‐mutant ECs, we first assessed the expression of the endogenous Notch target gene hairy and enhancer of split‐1 (*HES1*) in response to treatment with FGF7 in *FGFR2*‐mutant MFE280, MFE296 and AN3CA cells as well as WT FGFR2‐expressing Ishikawa and EM cells using qRT‐PCR. FGFR2 stimulation by FGF7 upregulated *HES1* mRNA levels in all *FGFR2*‐mutant cell lines (Figure 5A–C). *HES1* expression was significantly upregulated by up to 50‐fold after 24 h after FGF7 exposure in MFE280 (75.78 ± 3.71‐fold), MFE296 (12.96 ± 4.08‐fold) and AN3CA (61.3 ± 3.90‐fold), respectively. In contrast, no significant changes in *HES1* mRNA expression were observed in Ishikawa and EM cells (Figure S3A). Furthermore, none of the cell lines used in our study showed significant levels of *HES1* transcripts in the presence or absence of FCS supplementation (Figure S3B). These observations indicate that the induction of the Notch signalling pathway in response to FGF7 is *FGFR2*‐mutant EC cell type specific. Considering the previously described role of ADAM metalloproteases in the activation of Notch signalling pathways,^56^ we investigated the role of metalloprotease activity in the FGF7/FGFR2‐mediated induction of *HES1* and treated the *FGFR2*‐mutant EC cells with the metalloprotease inhibitor DPC. This treatment caused a significant downregulation of *HES1*, as determined by qRT‐PCR (Figure 5A–C) and a comparable decline in the *HES1* promoter activity, as determined by luciferase transactivation assays (Figure 5D–G). We next treated the *FGFR2*‐mutant EC cells with the γ‐secretase inhibitor DAPT, known to block proteolysis and transcriptional activation upon ligand binding,^57^ and assessed the FGF7‐dependent induction of *HES1* promoter‐driven luciferase activity in EM and EC cells (Figure 5D–G and Figure S3C). Treatment with DAPT had no significant impact on the already low *HES1* promoter‐driven luciferase activity in EM (Figure 5D) and Ishikawa EC cells (Figure S3C). In contrast, treatment with DAPT inhibited FGF7 induction of *HES1* promoter‐driven luciferase activity in all *FGFR2*‐mutant cell lines (Figure 5E–G). ADAM10 is a key component of the Notch signalling pathway^58^ and was significantly and differentially upregulated in *FGFR2*‐mutant ECs (Figure 3E). Although Notch signalling is not regulated through increasing the activity of ADAM10 but instead depends on ligand‐dependent exposure of the Notch cleavage site,^59^ increased ADAM10‐mediated shedding of other cell surface molecules has been implicated in multiple cancers.^60^, ^61^, ^62^, ^63^ To test whether ADAM10 activity is increased in *FGFR2*‐mutant EC cells, we stimulated these cells with increasing concentration of FGF7 (1–100 ng/mL) and monitored the release of the ADAM10 substrate BTC. EM cells (Figure 5H) as well as Ishikawa EC cells (Figure S3D) showed a very modest induction of BTC shedding and only at the highest FGF7 concentration, whereas FGF7 stimulation of *FGFR2*‐mutant EC cells caused a significant and dose‐dependent increase in the shedding of BTC (Figure 5I–K). These results indicate that ADAM10 activity is regulated by FGF7/FGFR2 signalling specifically in *FGFR2*‐mutant EC cancer cells.

To investigate whether FGF7 directly activated ADAM10 in *FGFR2*‐mutant EC cells or was dependent on prior ADAM17 activation, we used CRISPR*/*Cas9‐generated ADAM17‐deficient MFE280 (*MFE280^A17^* ^−/−^) cells. We stimulated these cells with the calcium ionophore ionomycin (IO), which was previously shown to activate ADAM10 and measured the shedding of BTC as a selective assay for ADAM10 activity.^34^, ^48^ We found that BTC was shed from *MFE280^A17^* ^−/−^ cells after IO stimulation while FGF7‐stimulated BTC shedding was decreased (Figure 5L). IO‐stimulated shedding of BTC was also sensitive to the ADAM10‐selective inhibitor GI254023X at a concentration that blocks ADAM10 but not ADAM17 (.2 µM^64^, ^65^), corroborating that IO‐induced shedding of BTC depends on ADAM10 in *MFE280^A17^* ^−/−^ cells (Figure 5M). Furthermore, the fold increase in BTC shedding from IO‐stimulated *MFE280^A17^* ^−/−^ cells was comparable with that of IO‐stimulated *MFE280* cells, suggesting that the presence of ADAM17 did not significantly influence the IO‐stimulated shedding of BTC (Figure 5N). Finally, FGF7‐induced shedding of BTC in *MFE280^A17^* ^−/−^ cells could be rescued by the overexpression of WT ADAM17 (Figure 5O). Collectively, these results suggest that NOTCH signalling is specifically upregulated in *FGFR2*‐mutant ECs in response to FGF7 stimulation and that ADAM17 is required for the FGF7‐induced activation of ADAM10‐mediated BTC shedding in *FGFR2*‐mutant EC cells.

### 3.7 Inhibition of ADAM17‐dependent signalling reduces FGF7‐stimulated anchorage independent growth of FGFR2‐mutant EC cells

**FIGURE 6:**
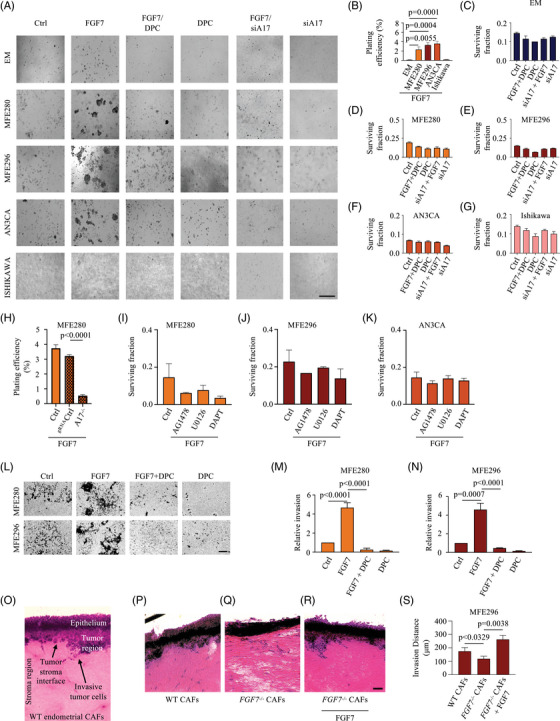
FGF7/Fibroblast growth factor receptor 2 (FGFR2)‐dependent activation of a disintegrin and metalloprotease 17 (ADAM17) is required for oncogenic growth of endometrial cancer (EC) cells harbouring mutations in the *FGFR2* in vitro: (A) anchorage‐independent growth was assessed by colony formation in soft agar. EM‐E6/E7/TERT (EM), MFE280, MFE296, AN3CA or Ishikawa cells were treated with or without FGF7 (50 ng/mL) in the presence or absence of DPC333 (DPC; 2.5 µM), CRM197 (10 µg/mL), AG1478 (10 µg/mL), small interfering RNA (siRNA) against ADAM17 (siA17; 20 pMol) or control siRNA (siCtrl, 20 pMol), as indicated. Images were taken after 2 weeks (scale bar: 500 µm); (B–G) plating efficiency (PE; number of colonies formed/number of cells seeded * 100%) (B) and surviving fraction; SF (number of colonies formed after treatment/number of cells seeded × PE × 100%) (C–G) of cells used in (A). Three independent experiments performed in duplicates. Colony counts from 10 random microscope fields in each replicate were analysed as % plating efficiency (B) and survival fraction (C–G). Data are expressed as mean ± standard error of mean (SEM); one‐way ANOVA with Dunnett's test. *p*‐Values indicate significantly increased plating efficiency of FGF7‐treated EC cells compared with EM cells; (H) PE of MFE280 cells transduced with guide RNA vector (^gRNA^Ctrl) or MFE280^A17−/−^ in the presence of FGF7. Three independent experiments performed in duplicates. Data are expressed as mean ± SEM; two‐way ANOVA with Tukey's multiple comparison test. *p*‐Value indicates a significant decrease in plating efficiency of FGF7‐treated ^gRNA^Ctrl MFE280 cells compared with *A17* ^−/−^ MFE280 cells; (I–K) quantification of anchorage‐independent growth of MFE280 (I), MFE296 (J) and AN3CA (K) cells treated with FGF7 (50 ng/mL) in the presence or absence of AG1478 (1 µM), U0126 (10 µM) or DAPT (10 µM); (L) representative images displaying invasive capabilities of MFE280 and MFE296 cells in transwell invasion assays. MFE280 and MFE296 were seeded in a transwell precoated with Matrigel and treated with FGF7 (50 ng/mL) in the presence or absence of DPC (2.5 µM); (M and N) relative invasion of MFE280 (M) and MFE296 (N). Three independent experiments performed in duplicates. Cell counts from four random microscope fields in each replicate were analysed for invasiveness (scale bar: 100 µm). Data are expressed as mean ± SEM; one‐way ANOVA with Tukey's multiple comparison test. *p*‐Values indicate significant differences in FGF7‐stimulated cell invasion compared to unstimulated (vehicle, Ctrl) or DPC‐treated cells; (O–S) invasion ability of MFE296 cells was determined using 3D‐organotypic co‐culture model system (O) by co‐culturing MFE296 cells with EC‐associated fibroblasts (P; wild type, WT CAFs) or *FGF7*‐deficient CAFs (Q and R; FGF7^−/−^ CAFs) in the presence or absence of 50 ng/mL FGF7 as indicated; (P–R) representative images of MFE296 cell invasion in the presence of CAFs alongside images of the tumour–stroma regions are shown. Cell counts from four random microscope fields in each replicate were analysed for invasiveness and expressed as average invasion distance (scale bar: 100 µm); (S) quantification of relative invasion distance in parts (P)–(R). *n* = 3, data are expressed as mean ± SEM; two‐way ANOVA with Tukey's multiple comparison test. *p*‐Values indicate a significant decrease in the cell invasion of MFE296 co‐cultured with *FGF7* ^−/−^ CAFs compared to FGF7‐treated or WT co‐cultures.

To further investigate the role of mutant FGFR2 on oncogenic cell growth, we performed clonogenic assays utilizing the EC cell lines described above. Concordant with a key role for FGF7‐driven oncogenicity, untreated *FGFR2*‐mutant EC cells formed only a few colonies, but treatment with FGF7 led to a significant increase of colony‐forming activity within 2 weeks (Figure 6A). Conversely, EM cells as well as *FGFR2*‐WT EC cells formed only a few colonies even in the presence of FGF7, suggesting that other factors drive anchorage‐independent cell growth in these cancer cells. Because ADAM17 is the subsequent step following FGFR2 activation in migration and proliferation of EM cells, targeting ADAM17 function in *FGFR2*‐mutant EC cells may have a beneficial effect in tumour suppression. To examine the therapeutic potential, we assessed colony formation in the presence of DPC (Figure 6A, a quantification of the results of three separate experiments is shown in Figure 6B–G). We found that the colony formation of *FGFR2*‐mutant EC cells was sensitive to DPC treatment, indicating that blockade of ADAM17 activity significantly inhibited anchorage‐independent growth in *FGFR2*‐mutant EC cells. Similarly, clonogenic activity could be inhibited by treatment with anti‐ADAM17 siRNA (Figure 6A, Figure S4A–D; a higher magnification of Figure 6A is shown in Figure S4E). Finally, clonogenic activity was also reduced in MFE280^A17−/−^ cells (Figure 6H and Figure S4F), further corroborating that the activation of ADAM17 by FGFR2 is critical for colony formation in *FGFR2*‐mutant EC cells. Although the blockade of ADAM17 reduced malignant colony formation in *FGFR2*‐mutant EC cells, it is not clear whether this result requires metalloprotease‐dependent EGFR‐mediated ERK activation and/or Notch activity. To determine the functional relevance of these two signalling pathways, we monitored the impact of EGFR/ERK and Notch blockade on the clonogenic phenotype of FGF7‐stimulated *FGFR2*‐mutant EC cells compared with *FGFR2*‐WT EC cells. To this end, FGF7‐stimulated *FGFR2*‐mutant EC cells were either treated with AG1478, U0126, a highly selective inhibitor of both MEK1 and MEK2, or treated with DAPT. A strong inhibition of FGF7‐stimulated colony formation by treatment with AG1478, U0126 or DAPT was observed in MFE280 cells (Figure 6I and Figure S4G). Treatment of MFE296 and AN3CA cells with AG1478, U0126 or DAPT also strongly reduced FGF7‐stimulated colony formation, suggesting that both EGFR and Notch‐dependent signalling pathways drive the colony formation of *FGFR2*‐mutant EC cells (Figure 6J,K and Figure S4G).

Next, we examined the effect of FGF7‐mediated FGFR2 activation on *FGFR2*‐mutant EC cell migration and invasion (Figure 6L; a higher magnification of Figure 6L is shown in Figure S4H). Using a Matrigel‐coated transwell assay, we found that FGF7 significantly increased the ability of MFE280 cells to migrate and invade through Matrigel, whereas DPC significantly reduced the FGF7‐induced invasive capacity (Figure 6L, top row, a quantification of the results of three separate experiments is shown in Figure 6M). Similar results were observed in MFE296 cells (Figure 6L, bottom row, a quantification of the results of three separate experiments is shown in Figure 6N), altogether suggesting that DPC not only reduced oncogenic cell growth but also inhibited metastatic capacity in vitro.

Previous studies have shown that FGF7 is produced by stromal fibroblasts.^66^ To determine the potential effects of fibroblast‐released FGF7 in EC, we investigated the influence of EC patient‐derived cancer‐associated fibroblasts (CAFs^67^) on the migration of invasive *FGFR2*‐mutant EC cells. We first validated for the expression of FGF7 in CAFs. qRT‐PCR demonstrated that FGF7 was indeed expressed significantly in CAFs corroborating prior studies (Figure S4I).^68^, ^69^ Then we utilized a 3D tumour–stromal organotypic co‐culture model (Figure 6O) and characterized the invasive phenotype of MFE296 cells in co‐culture with either WT or CRISPR*/*Cas9‐edited FGF7‐deficient (FGF7^−/−^) CAFs. We found a significant decrease in MFE296 cell migration distance for *FGF7* ^−/−^ CAFs when compared with WT CAF co‐cultures (Figure 6P,Q). Notably, the decrease in cell invasion could be rescued by the addition of recombinant FGF7 (Figure 6R, a quantification of the results of three separate experiments is shown in Figure 6S), suggesting that stromal fibroblasts are a key source of FGF7 which could influence the migratory profiles of *FGFR2*‐mutant EC cells.

To evaluate the causative role of *FGFR2* mutations in an isogenic background, we deleted *FGFR2* in EM (EM*^FGFR2^* ^−/−^) cells and compared the effects of overexpressed FGFR2‐mutants (S252W and N550K, respectively) with overexpressed FGFR2‐WT in cell‐based shedding assays and found that shedding of HB‐EGF in FGFR2‐mutant expressing EM*^FGFR2^* ^−/−^ cells was more sensitive to FGF7 stimulation when compared to FGFR2‐WT expressing EM*^FGFR2^* ^−/−^ cells (Figure S5A and B). To further demonstrate the functional consequences of mutant FGFR2, we performed colony formation assays and found that the overexpression of the FGFR2‐mutants in EM*^FGFR2^* ^−/−^ cells supports anchorage‐independent growth and potentially drives transformation and oncogenic growth of EM cells (Figure S5C). Finally, we found that Notch signalling pathway components, including *JAG2* and *DLL3* transcripts, were differentially upregulated in FGFR2‐mutant overexpressing EM*^FGFR2^* ^−/−^ cells (Figure S5D).

### 3.8 Targeting ADAM17 inhibits tumour progression in NSG mice

**FIGURE 7:**
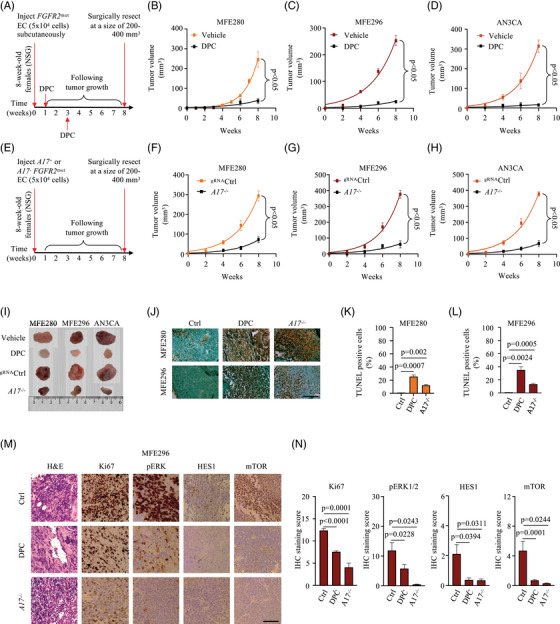
Inactivation of a disintegrin and metalloprotease 17 (ADAM17) reduces *fibroblast growth factor receptor 2* (*FGFR2*)‐mutant endometrial tumour growth in vivo: (A) dose, route and schedule are shown; (B–D) therapy of NOD‐scid mice injected subcutaneously with MFE280 (B), MFE296 (C) or AN3CA (D) and treated with DPC333 (DPC); (E–H) therapeutic effect of ADAM17 inactivation against MFE280 (F), MFE296 (G) or AN3CA (H) tumour xenografts; (I) typical photos of tumours on day 56 for the experiments shown in parts (B)–(D) and (F)–(H). Two independent experiments with *n* = 6 mice per group. Data are expressed as mean ± standard error of mean (SEM); two‐way ANOVA showed a significant main effect of DPC (B–D) and ADAM17 inactivation (F–H), respectively; (J) immunohistochemical staining of xenograft endometrial cancer tissues to examine the induction of apoptosis using TUNEL assay. One representative of two independent experiments performed with *n* = 6 mice is shown; (K and L) quantitation of apoptotic cells from TUNEL staining. Cell counts from 10 random microscope fields in each tumour section were analysed as % apoptotic (TUNEL‐positive) cells. Data are expressed as mean ± SEM; one‐way ANOVA with Dunnett's test. *p*‐Values indicate a significant increase in the percentage of TUNEL‐positive cells of vehicle‐treated tumours compared to DPC‐treated or *ADAM17*‐deficient tumours; (M) haematoxylin and eosin (H&E) staining of paraffin sections of endometrial xenograft tumours and expression levels of pERK1/2, HES1, mTOR and proliferation marker gene, Ki67, assessed by immunohistochemistry staining (scale bar: 100 µm). Representative images of two independent experiments performed with *n* = 6 mice is shown; (N) IHC staining scores of (M). A total of 10 random microscope fields in each tumour section were scored and expressed as % apoptotic (positive) IHC staining scores. Data are expressed as mean ± SEM; one‐way ANOVA with Dunnett's test. *p*‐Values indicate a significant higher IHC staining score of untreated tumours compared to DPC‐treated or *ADAM17*‐deficient tumours.

We next evaluated the in vivo antitumor efficacy of ADAM17 inhibition in a murine xenograft tumour model. Subcutaneous implantation of MFE280, MFE296 or AN3CA cells into immunodeficient NOD.Cg‐*Prkdc^scid^ Il2rg^tm1Wjl^*/SzJ (NSG) mice resulted in the growth of solid tumours of approximately 100–300 mm^3^ within 6 weeks that continued to increase in size until the humane end point of the experiment. Administration of DPC after 1 and 3 weeks significantly inhibited xenografted tumour volume in all treated groups when compared to vehicle‐treated mice (Figure 7A–D). Tumour volume was also reduced in tumours derived from ADAM17‐deficient FGFR2‐mutant EC cells (MFE280^A17−/−^, MFE296^A17−/−^, AN3CA^A17−/−^, Figure 7E–H), further corroborating that ADAM17 activity is critical for tumour growth in *FGFR2*‐mutant EC cells (Figure 7E–H). Furthermore, the inhibition and genetic deletion of ADAM17 resulted in a significant reduction of xenografted tumour size and weight in all experimental mice (Figure 7I and Figure S6A–C).

Previous studies have shown that the genetic deletion of ADAM17 induces apoptosis due to the suppression of EGFR signalling,^70^ suggesting that DPC‐mediated growth inhibition may involve apoptosis. In agreement with this, histological sections from *FGFR2*‐mutant ECs showed that there was a significant increase in apoptosis (as indicated by brown‐stained nuclei) following DPC injection, compared to that of untreated tumours (Figure 7J–L). A similar increase in apoptosis was also observed in tumours derived from ADAM17‐deficient FGFR2‐mutant EC cells (Figure 7J, a quantification of the results of three separate experiments is shown in Figure 7K,L; and a higher magnification of Figure 7J is shown in Figure S6D).

Ki‐67, a nuclear nonhistone protein, is one of the major markers of tumour proliferation^71^ used as a decision‐making tool for adjuvant therapy.^72^ The immunohistochemical assessment of tumour proliferation showed higher Ki‐67 in the control group as compared with the DPC‐treated group (Figure 7M; a higher magnification of Figure 7M is shown in Figure S6E). In our in vitro analysis, we found that mutant FGFR2‐modulated the expression of Notch and EGFR in *FGFR2*‐mutant EC cells. Corroborating the in vitro findings, the tumours from the DPC‐treated mice exhibited reduced pERK as well as lower expression of HES1 and mTOR (Figure 7M,N). A similar decrease in HES1 and mTOR expressions was also observed in tumours derived from ADAM17‐deficient MFE296 cells (Figure 7M,N). Collectively, these results show that the inactivation of ADAM17 results in the suppression of tumour growth, inhibition of cellular proliferation and increased apoptosis in *FGFR2*‐mutant ECs.

## 4 DISCUSSION

Next‐generation sequencing has identified various mutations in *FGFR2* in multiple cancers, including EC,^73^ and FGFR2 has been suggested as a novel therapeutic target.^8^, ^74^, ^75^ Although these studies have established a key role for FGFR2 in EC,^4^, ^6^, ^7^ the causal relationship between these mutations and tumourigenesis is not well understood. In this study, we investigated the role of mutant FGFR2 in the activation of ADAM17‐mediated EGFR/ERK signalling in several human EC cell lines as a representative pre‐clinical model for EC. Using gain‐ and loss‐of‐function studies together with pharmacological approaches, we show that in normal EM cells, FGFR2 drives cell migration and proliferation by activating ADAM17 and the subsequent release of HB‐EGF, which in turn activates ERK via the EGFR signalling pathway. In contrast to WT FGFR2, the expression of EC‐linked mutant FGFR2 resulted in a heightened sensitivity of EC cells to FGF7 stimulation. Our results suggest that low FGF7 levels, of which endometrial stromal cells are a rich source,^68^ trigger the activation of ADAM17 and downstream signalling of the EGFR leading to increased oncogenic growth. Intriguingly, FGF7‐induced FGFR2 activation also resulted in Notch signalling in *FGFR2*‐mutant EC cells. Notably, this mechanism was unique to *FGFR2* mutations as normal EM cells as well as WT FGFR2‐expressing EC cells did not engage in Notch activation and downstream signalling upon FGF7 stimulation.

The majority of single‐nucleotide variants in FGFR have been reported to occur in *FGFR2* and are found at high frequencies in EC and are associated with poor outcomes.^76^ Our FGFR2 mutation analysis in a series of 62 women visiting the Cancer Center at the University of Iowa combined with that of 402 TCGA cases revealed, in accordance with others,^4^, ^6^, ^77^ that the overall *FGFR2* mutation rate in the combined cohort of 464 EC cases was approximately 10% (53/464), whereas nearly a quarter (24.2%) of our patients with EC carried 1 of the *FGFR2* mutations (15/62). Ethnic, racial and/or regional differences may explain in part the higher rate of mutations in our patient population. Interestingly, differences in the distribution of oncogenic mutations have been well documented for gynecologic cancers, including *breast cancer genes* (*BRACA*) *1* and *BRACA 2*.^78^, ^79^ The knowledge about the presence and prevalence of mutations in specific populations could be of importance for selecting women eligible for *FGFR2* analysis and will greatly facilitate the detection of mutations.

Previous studies have reported on the constitutive and ligand‐independent activation of FGFR2, which was due to the constitutive phosphorylation of the FGFR2 kinase domains.^6^ Surprisingly, our findings indicate that, in the absence of FGF7, *FGFR2*‐WT as well as *FGFR2*‐mutant EC cells need to adhere to a solid matrix to remain viable and proliferate. However, *FGFR2*‐mutant EC cells stimulated with FGF7 lost this requirement, which resulted in anchorage independence and in their ability to form dense, raised foci as well as proliferating colonies when suspended in a semisolid agar, suggesting that these processes depend on FGF7/FGFR2‐mediated activation of ADAM17. Although the root of these divergences remains unknown, it is possible that different culturing and medium conditions, specifically with unintended FGFR2‐stimulating supplementation, might have been sufficient to induce FGFR2 activation in previous studies. Additionally, those results could possibly be due to the high protein expression of ectopically overexpressed FGFR2, potentially leading to elevated levels of tyrosine kinase activity following artificial receptor dimerization.

It is notable that none of the tested FGFR2 mutants affected the constitutive activity of ADAM17. These results suggest that responsiveness to agonist stimulation, in addition to its constitutive activity, might be required for mutant FGFR2‐dependent oncogenic activity at least in the context of ADAM17 activation. Although FGF7 expression is upregulated in many cancers,^80^ it is expressed at lower levels in EC by comparison with the corresponding normal tissue.^81^ Our demonstration that mutant FGFR2 enhances the sensitivity to FGF7‐induced ADAM17 activity provides an explanation of how these tumours might adapt in an environment with reduced growth factors. Collectively, these findings suggest that the oncogenic potential of the FGFR2 mutants might arise due to an increased affinity and/or sensitivity of the mutant receptor for the FGF7 ligand, which leads to the activation of ADAM17 under conditions where the availability of ligand is limiting.

Our data not only demonstrated that *FGFR2*‐mutations require less FGF7 stimulation to activate ADAM17 but also result in the activation of EGFR and Notch signalling pathways as well as in the activation of ADAM10 in an ADAM17‐dependent manner. To our knowledge, this is the first disease context in which ADAM10 activation has been shown to be dependent on ADAM17 function. Although the molecular mechanisms of this crosstalk are not known, it is possible that the aberrant activation of ADAM17/EGFR‐mediated downstream signalling pathways in *FGFR2*‐mutant ECs leads to an increased proteolytic activity of ADAM10 or alternatively increased accessibility to its substrate.^82^ ADAM10 and ADAM17 are part of distinct multiprotein/substrate complexes.^82^, ^83^ In addition, previous studies demonstrated that proteases can modulate the activity of other proteases through prodomain cleavage.^84^ Therefore, it is also possible that aberrant complex formation in *FGFR2*‐mutant ECs leads to the direct interaction of ADAM17 with ADAM10 and the subsequent modification of its proteolytic activity. Although we show that FGF7 induced the activation of ADAM10, we also show that FGF7 also induced the expression of Notch receptors and ligands. The increase in Notch‐signalling could be due to the increase in ligand‐dependent exposure of the Notch cleavage site or an increase in accessibility by ADAM10 as previous studies have shown that an increase in ADAM10 activity alone does not increase Notch processing.^59^ Future studies will be needed to determine how these metalloproteases are activated in response to *FGFR2* mutations and will be key to illuminating the best therapeutic approaches to treating EC patients with *FGFR2* mutations. Given the altered sensitivity of various EC‐linked *FGFR2* mutations to FGF7 and ADAM17/ADAM10 activation, it is conceivable that metalloprotease blockade may be a more effective therapeutic strategy than nonselective receptor tyrosine kinase inhibitors alone.

Our results also show that the activation of ADAM17 by FGFR2 depends on Src, PI3‐kinase and p38 MAP‐kinase. Previous studies have shown that Src, which can activate ADAM17,^65^ and PI3K are important for FGF7‐stimulated migration,^85^ suggesting that this also depends on the activation of ADAM17. Whether mutations in *FGFR2* alter the activation of these downstream effectors leading to ADAM17 activation in EC remains to be tested. Notably, RNA‐sequencing results from EC cells indicated that EGFR, PI3K, NOTCH2 and NOTCH3 were prominently upregulated by *FGFR2* mutations. All four of these genes have been linked with EC,^86^, ^87^, ^88^, ^89^, ^90^, ^91^, ^92^, ^93^ as well as with FGFR2 function.^9^, ^94^, ^95^, ^96^, ^97^, ^98^ These results suggest that *FGFR2* mutations could regulate core transcriptomic networks that endow EC with further oncogenic properties. Mechanistically, FGFR2‐mediated and ADAM17‐dependent EGFR signalling could allow for the integration of Notch signalling via chromatin remodelling events.^99^, ^100^, ^101^ Alternatively, bidirectional crosstalk between EGFR and Notch or additive transcriptional pathways regulated individually via EGFR and Notch could result in enhanced oncogenic potential.^102^

## 5 CONCLUSIONS

**FIGURE 8:**
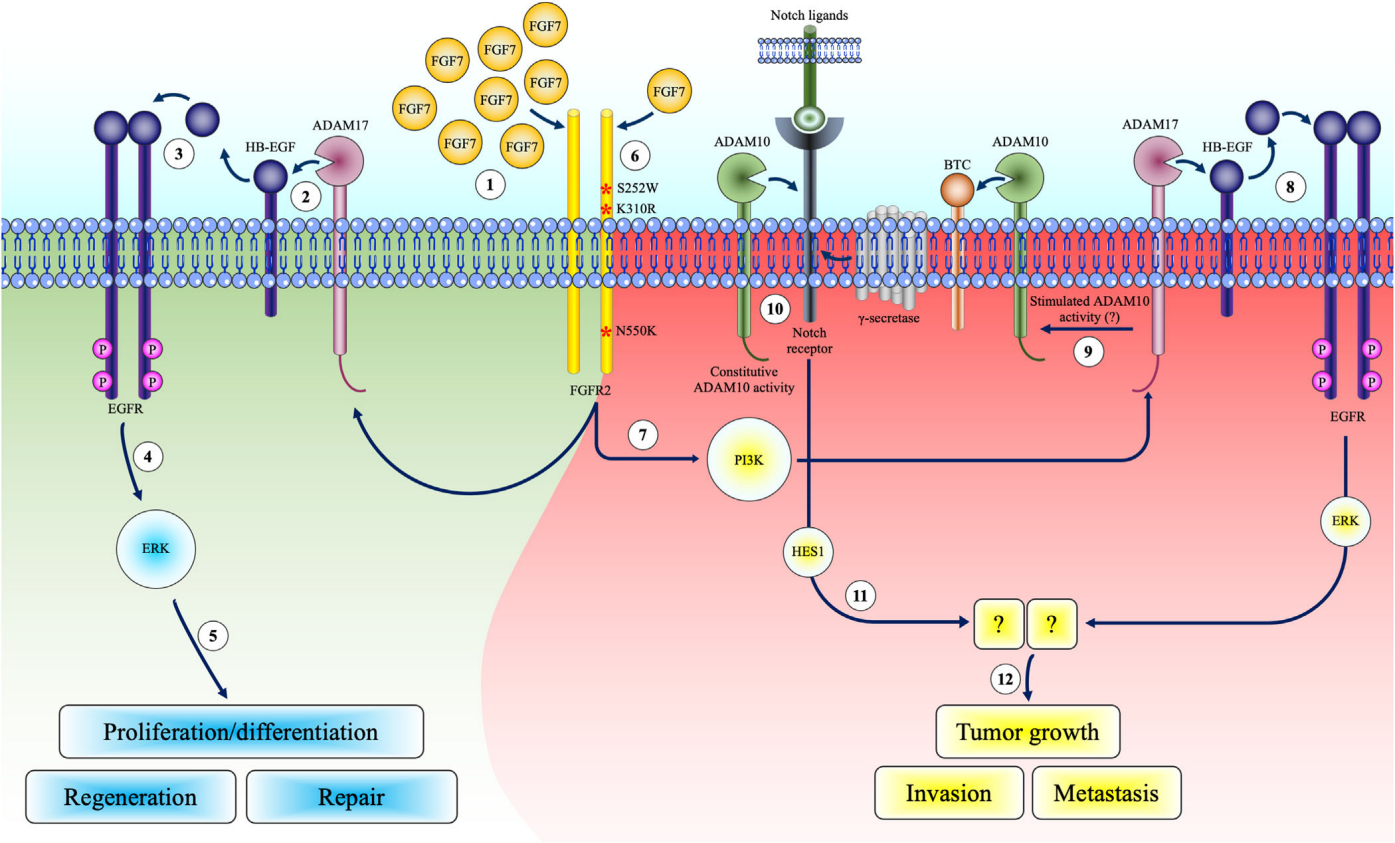
Fibroblast growth factor receptor 2 (FGFR2) mutation‐mediated activation of a disintegrin and metalloprotease 17 (ADAM17) promotes endometrial cancer (EC) progression via dual engagement of epidermal growth factor receptor (EGFR) and Notch signalling pathways. (Left) In normal endometrial cells (green) with wild type fibroblast growth factor receptor (FGFR) 2, binding of FGF7 to FGFR2 (1) stimulates ADAM17‐mediated release of membrane‐bound EGFR‐ligand heparin‐binding EGF‐like growth factor (HB‐EGF) (2), which activates EGFR (3) and extracellular signal‐regulated kinase (ERK) (4), thereby promoting a variety of biological functions, including the positive regulation of tissue development, repair and regeneration (5). (Right) In cells expressing mutant FGFR2 (red), normal FGFR2 function is disrupted, resulting in ADAM17‐dependent oncogenic growth. FGFR2 mutations (6) significantly enhance the sensitivity to FGF7‐mediated activation of ADAM17 (7) and subsequent transactivation of the EGFR (8). FGFR2 mutations also trigger the activation of ADAM 10‐ and γ‐secretase‐mediated Notch signalling in an ADAM17‐dependent manner (9,10), subsequently activating transcription of hairy and enhancer of split (HES1) and perhaps repressors of cell cycle inhibitors (11), allowing for tumour initiation and metastatic progression (12).

Our findings that EC‐linked mutations in FGFR2 increase ADAM17 activity provides a well‐defined description of a molecular mechanism associated with these pathogenic mutations, implicates increased ADAM17 activity in tumour formation and identifies a new druggable target in EC. Further, our findings demonstrate that aberrant FGFR2 activity elicits increased ADAM17 function and gene dysregulation and implies a novel mechanistic basis for oncogenic transformation through transcriptional reprogramming. Finally, our discovery of increased Notch and EGFR/PI3K signalling in *FGFR2*‐mutant EC suggests a novel route to oncogenic transformation and a viable target for pharmacologic intervention. Taken together, our results support the hypothesis that FGFR2 crosstalk has a dual role in the endometrium, by regulating cell proliferation in normal endometrium, but acting as an oncogene in endometrial carcinoma (Figure 8).

## CONFLICT OF INTEREST STATEMENT

The authors declare that they have no conflict of interest.

## AVAILABILITY OF SUPPORTING DATA

All data supporting this study and its findings are available within the article. Datasets for the prediction model have been deposited in Gene Expression Omnibus (GEO) database at National Center for Biotechnology Information (NCBI)website: https://www.ncbi.nlm.nih.gov/geo/. Datasets with RNA‐seq can be browsed by their accession number: GSE156699. The validation part of this study was performed in silico, with de‐identified publicly available data. All data from TCGA is available at their website: https://portal.gdc.cancer.gov/. Software utilized by this study is also publicly available at Bio‐conductor website: http://bioconductor.org/.

## Supporting information

Supporting Information

Supporting Information
